# Chinese herbal medicine-derived exosome-like nanovesicles for orthopaedic diseases: evidence, methods, and translation

**DOI:** 10.3389/fcell.2026.1853729

**Published:** 2026-07-10

**Authors:** Yi Hang Zhu, Jia Hao Wang, Xiao Jun Zhang, Meng Cheng Zhu, Guo Hua Wang, Yan Ming Hao

**Affiliations:** 1 Department of Orthopedics, Affiliated Kunshan Hospital of Jiangsu University, Suzhou, Jiangsu, China; 2 Department of Orthopedics, The First People’s Hospital of Kunshan, Gusu School, Nanjing Medical University, Suzhou, Jiangsu, China; 3 The Fourth People’s Hospital of Kunshan, Suzhou, Jiangsu, China; 4 The Fifth People’s Hospital of Kunshan, Suzhou, Jiangsu, China

**Keywords:** Chinese herbal medicine, orthopaedic diseases, osteoarthritis, osteoporosis, plant-derived exosome-like nanovesicles, translational perspectives

## Abstract

Chinese herbal medicine/medicinal plant-derived exosome-like nanovesicles (CHM/medicinal plant-derived ELNVs) are emerging as promising nanoplatforms for orthopaedic diseases because of their favorable biocompatibility, complex bioactive cargos, and potential multi-target regulatory activity. This mini-review summarizes current evidence with emphasis on a minimum reporting framework, disease-module interpretation, mechanistic integration, and translational barriers. Methodologically, future studies should clearly report source authentication, isolation strategy, vesicle characterization, purity or cargo fingerprints, route-specific safety indicators, and disease-relevant potency readouts. In terms of disease evidence, osteoporosis currently represents the most mature application module, whereas osteoarthritis and bone repair are emerging local-delivery fields, and intervertebral disc disorders, tendon/ligament injury, and rheumatoid arthritis-related musculoskeletal inflammation remain early or indirect indications. Mechanistically, CHM/medicinal plant-derived ELNVs may regulate bone remodeling, bone–vascular coupling, inflammation, oxidative stress, and extracellular matrix homeostasis through coordinated cargo- and delivery-dependent effects. However, meaningful translation is still limited by insufficient CMC-oriented batch control, inadequate cargo-causality validation, insufficient pharmacokinetic and biodistribution evidence, and limited long-term safety data. Overall, CHM/medicinal plant-derived ELNVs remain promising but require reproducible, route-specific, and clinically interpretable evidence chains.

## 1 Introduction

Orthopaedic disorders, particularly osteoporosis and osteoarthritis, are increasing with population aging and impose a growing clinical and socioeconomic burden. Their progression is rarely driven by a single pathway; instead, it usually reflects the convergence of inflammation, oxidative stress, extracellular matrix (ECM) remodeling, impaired bone remodeling, and altered bone–vascular coupling (Bai et al., 2024; Zeng et al., 2024). This multi-pathway nature has stimulated interest in therapeutic platforms capable of coordinating multiple biological processes rather than targeting isolated molecular events.

**FIGURE 1:**
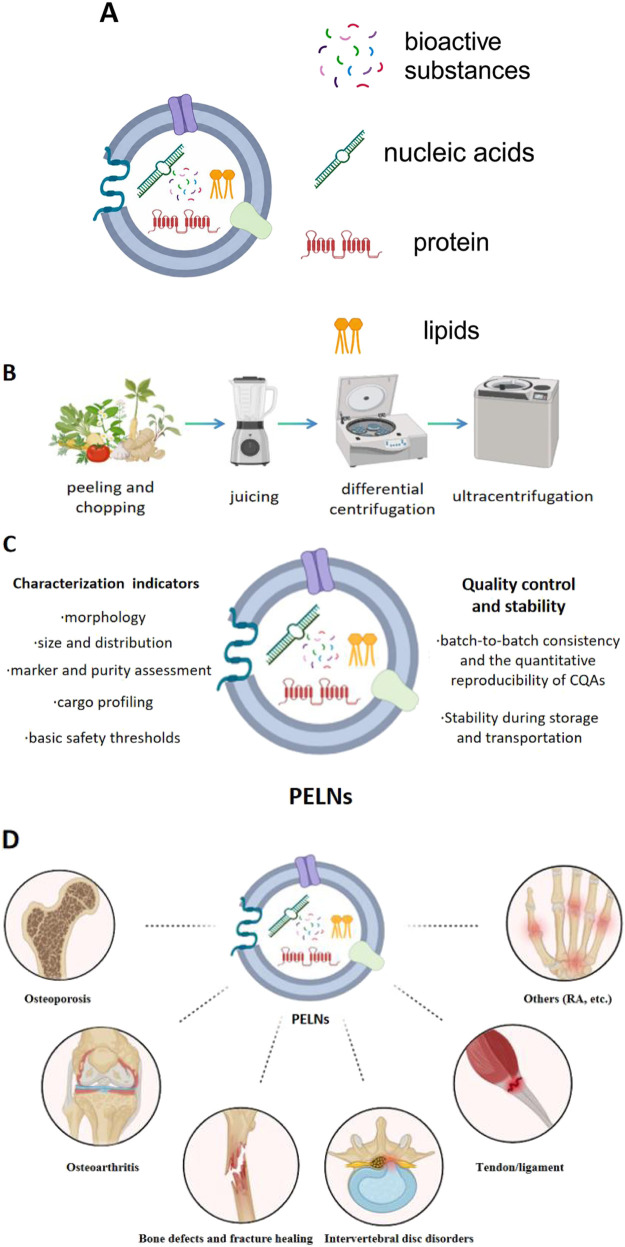
Overview of plant-derived exosome-like nanovesicles and their potential applications in orthopaedic diseases. **(A)** Main components of PELNs, including nucleic acids, proteins, lipids, and small bioactive substances, which together contribute to their biological functions and therapeutic potential. **(B)** General workflow for the isolation of PELNs. **(C)** Characterization and quality-control considerations for PELNs. **(D)** Overview of the potential applications of PELNs in orthopaedic diseases, including osteoporosis, osteoarthritis, bone defects and fracture healing, intervertebral disc degeneration, tendon injury, and rheumatoid arthritis.

Extracellular vesicles (EVs) have emerged as endogenous-like mediators of intercellular communication through the transfer of nucleic acids, proteins, lipids, and other bioactive molecules, which are also major cargo components of PELNs (Lo et al., 2024) (Figure 1A). Among them, plant-derived exosome-like nanovesicles (PELNs) have attracted increasing attention because of their scalable sourcing, relatively low immunogenicity, favorable biocompatibility, and potential suitability for repeated or long-term administration (Ali et al., 2022; Mu et al., 2023; Zhu et al., 2024). In musculoskeletal contexts, accumulating preclinical evidence suggests that PELNs may influence osteogenesis–osteoclastogenesis balance, inflammatory and immune microenvironments, antioxidant responses, and cartilage or bone matrix homeostasis (López de las Hazas et al., 2023; Jin et al., 2024; Lv et al., 2024).

Chinese herbal medicine and medicinal plant-derived exosome-like nanovesicles (CHM/medicinal plant-derived ELNVs) represent a particularly relevant subgroup within this field. Their value lies not only in their plant origin, but also in their potential to preserve source-related pharmacological complexity, phytochemical-associated cargo features, and disease-aligned multi-target regulatory activity. These characteristics may be especially meaningful for orthopaedic disorders, in which chronic degeneration and tissue repair are shaped by interacting inflammatory, metabolic, vascular, and matrix-related signals.

However, current evidence remains heterogeneous. Differences in source authentication, isolation strategy, vesicle characterization, cargo identification, delivery route, and functional readouts limit cross-study comparability and translational interpretation (Wang et al., 2023; Zhao et al., 2024). Therefore, this mini-review focuses on CHM/medicinal plant-derived ELNVs in orthopaedic diseases and summarizes current evidence along three linked dimensions: methodological reporting, disease-module evidence, and translational barriers. Evidence from conventional edible plant-derived EVs or engineered mammalian EV platforms is cited only as supportive biological or delivery-design analogy, rather than as direct efficacy evidence for CHM/medicinal plant-derived ELNVs.

## 2 Methodological essentials: a minimum reporting framework

For CHM/medicinal plant-derived ELNVs to be interpreted reproducibly in orthopaedic studies, methodological reporting should be concise but sufficiently complete. Rather than expanding into a full technical review of isolation and characterization, future studies should provide a minimum reporting framework that links source, product identity, safety, and disease-relevant function. This framework is particularly useful for mini-review interpretation because it allows heterogeneous studies to be compared without overemphasizing technical details.

First, source authentication should be clearly described, including plant species, medicinal part, origin, cultivation or processing conditions, and batch information. This is particularly important for CHM/medicinal plant-derived ELNVs because plant growth conditions, harvest season, and processing procedures may substantially affect vesicle yield and cargo composition. Second, the isolation strategy should be reported in enough detail to allow cross-study comparison, including key steps such as homogenization, juicing, centrifugation, filtration, density-gradient purification, size-exclusion chromatography, or other enrichment methods; a general isolation workflow is shown in Figure 1B. Yield and storage conditions should also be stated when available (Zhao et al., 2024; Mun et al., 2025).

Third, vesicle morphology and particle size should be verified using complementary approaches. Transmission electron microscopy or atomic force microscopy can support membrane-associated morphology, whereas nanoparticle tracking analysis or dynamic light scattering can provide particle size and distribution data (Jin et al., 2024; Langellotto et al., 2024; Wu et al., 2024). Fourth, because plant-derived preparations may contain co-isolated phytochemicals, protein aggregates, membrane fragments, or other nanosized particles, purity assessment and cargo fingerprinting are essential. In the absence of universally accepted plant-vesicle markers, proteomic, lipidomic, small-RNA, and phytochemical-associated profiles may help define product identity and batch comparability (Hao et al., 2024; Temviriyanukul et al., 2024). These characterization and quality-control elements are summarized in Figure 1C.

Fifth, route-specific safety indicators should be aligned with the intended orthopaedic application. Oral studies should consider gastrointestinal stability, tolerability, exposure–response relationships, and systemic distribution, whereas intra-articular or defect-site delivery requires stricter evaluation of sterility, endotoxin burden, local inflammatory reactions, and tissue retention (Fang and Liu, 2022; Kim et al., 2022; Zhao et al., 2024). Finally, each study should include at least one disease-relevant potency readout. For osteoporosis or bone repair, this may involve osteogenesis–osteoclastogenesis balance or angiogenesis–osteogenesis coupling; for osteoarthritis or disc degeneration, paired ECM synthesis–degradation markers and inflammatory or oxidative-stress readouts are more appropriate (Cao et al., 2024; Feng et al., 2024; Huang et al., 2025). This minimum framework allows methodological evaluation to support, rather than overshadow, disease-oriented interpretation.

## 3 Orthopaedic disease modules

Orthopaedic disorders share several pathological features, including chronic inflammation, oxidative stress, extracellular matrix (ECM) imbalance, impaired bone remodeling, and altered tissue repair responses. These overlapping mechanisms provide a rationale for evaluating CHM/medicinal plant-derived ELNVs within a disease-module framework rather than as single-pathway interventions (Xia et al., 2025). In this review, the disease evidence is organized according to relative maturity: osteoporosis as the leading evidence module, osteoarthritis and bone repair as emerging local-delivery modules, and intervertebral disc disorders, tendon/ligament injury, and rheumatoid arthritis-related musculoskeletal inflammation as early or indirect indications (Figure 1D). Within this framework, reported efficacy should be interpreted according to material identity, mechanism-linked functional readouts, route-appropriate delivery feasibility, and translational depth (Mun et al., 2025). The relative evidence maturity, key readouts, main gaps, and translational priorities of each orthopaedic disease module are comparatively appraised in Table 1, with attention to model support, methodological characterization, PK/BD availability, cargo-causality validation, and translational depth.

**TABLE 1:** Comparative appraisal of evidence maturity and translational readiness of CHM/medicinal plant-derived ELNVs across orthopaedic disease module.

Disease module	Evidence maturity	Key readouts	Main gaps	Translational priority
Osteoporosis	Relatively highest	Osteogenesis osteoclastogenesis balance; RUNX2/OCN; mineralization; RANKL/OPG; gut-bone or immune-metabolic signals	Variable characterization; incomplete cargo causality, PK/BD, dosing, and endpoint standardization	Priority module for standardized preclinical validation; strongest current orthopaedic evidence base
Osteoarthritis	Early to moderate	ECM synthesis-degradation balance; MMP-13/ADAMTS- 5; COL2A1/Aggrecan; inflammatory cytokines; structural or pain endpoints	Limited direct CHM/medicinal plant-derived ELNV evidence; insufficient intra-articular retention, local safety, PK/BD, and cargo causality	Promising local-delivery field, but disease-modifying claims should remain cautious
Bone defect/fracture healing	Moderate and emerging	Angiogenesis-osteogenesis coupling; RUNX2/OCN; CD31/endomucin or H-type vessel signals; mineralized tissue formation	Incomplete long-term remodeling data, scaffold/product characterization, full PK/BD, and cargo-function validation	Strong rationale for biomaterial-assisted local regeneration studies
Intervertebral disc disorders	Low	ECM homeostasis; MMP- 13/ADAMTS-5; COL2A1/Aggrecan; oxidative or mitochondrial stress; imaging and mechanical assessment	Direct evidence is scarce; intradiscal retention, penetration, local safety, PK/BD, and cargo causality are not established	Exploratory, hypothesis- generating direction
Tendon/ligament injury	Low	Collagen I/III balance; scar formation; inflammatory control; load-bearing and biomechanical recovery	Direct CHM/medicinal plant- derived ELNV evidence is lacking; mechanical-loading validation, retention kinetics, and long-term functional endpoints are insufficient	Future exploratory module requiring mechanically relevant models
RA-related musculoskeletal inflammation	Early to moderate	Synovial inflammation; macrophage M1/M2 polarization; TNF- 0, IL-1 B, IL-6; joint swelling or arthritis scores	More immunomodulatory than structural-regenerative; limited long-term safety, PK/BD, and cargo-specific mechanisms	Relevant inflammatory musculoskeletal module; translational claims should be conservative

### 3.1 Osteoporosis as the leading evidence module

Osteoporosis is currently the most mature orthopaedic application context for CHM/medicinal plant-derived ELNVs. Available studies support two main directions: direct regulation of the osteogenesis–osteoclastogenesis balance and indirect modulation of the gut–immune–metabolic axis (Słupski et al., 2021; Han R. et al., 2025; Xia et al., 2025). Yam-derived vesicles and Morinda officinalis-derived EV-like particles have been linked to osteogenic differentiation, mineralization, and improved bone parameters through BMP-2/p-p38/RUNX2-and MAPK-CREB/RSK1-related signaling, whereas Pueraria lobata-derived vesicles suggest a microbiome–metabolite–bone axis involving autophagy and trimethylamine N-oxide regulation (Hwang et al., 2023; Zhan et al., 2023; Cao et al., 2024).

Bone repair-oriented evidence also provides supportive cues for osteoporosis-related translation, especially when osteogenesis is combined with local retention strategies. Sea buckthorn-derived vesicles incorporated into GelMA hydrogels have been associated with aau-miR168/LBH/RUNX2-mediated osteogenic repair, while Cissus quadrangularis callus systems suggest a potentially scalable plant-cell-engineering route for vesicle production (Gupta et al., 2023; Seo et al., 2023; Zhao et al., 2025). Non-CHM systems, such as oyster mantle-derived exosomes, may provide endpoint or pathway comparison, but require CHM-specific validation before extrapolation (Fang et al., 2024; Hu et al., 2024). Overall, OP shows the strongest preclinical support among current orthopaedic modules, although dosing regimens, identity criteria, endpoint panels, and PK/BD evidence remain heterogeneous.

### 3.2 Osteoarthritis and bone repair as emerging local-delivery modules

Osteoarthritis and bone defect/fracture healing represent promising but less mature application settings, with both fields emphasizing local exposure, retention, and disease-aligned functional readouts. OA is mechanistically suitable for CHM/medicinal plant-derived ELNV exploration because inflammation, oxidative stress, and cartilage ECM imbalance interact throughout disease progression (Mao et al., 2021; Yin et al., 2022; Qian et al., 2024). Current evidence mainly supports anti-inflammatory, antioxidant, and ECM-protective phenotypes rather than robust disease-modifying efficacy in established OA models. For example, Perilla-derived vesicles have been reported to reduce ROS and inflammatory mediators in zebrafish and inflammatory models (Huang et al., 2025; Yang et al., 2025). Future OA studies should therefore prioritize paired ECM synthesis–degradation readouts, intra-articular retention, local safety, and structural or pain-related endpoints.

Bone defects and fracture healing provide a more regeneration-oriented local-delivery scenario. The key translational logic is not only intrinsic bioactivity, but also spatial retention and temporal alignment between early angiogenesis and later osteogenesis, especially in hydrogel- or scaffold-assisted systems (Zhao et al., 2025). Evaluation should combine angiogenic and osteogenic readouts, such as CD31/endomucin or H-type vessel-related signals together with RUNX2 or osteocalcin (OCN). Non-CHM or engineered vesicle systems may offer useful design principles, including scalable carrier design, pH-responsive release, chondrocyte- or tissue-affinity targeting, and sustained local exposure, but these should be interpreted as delivery analogies rather than direct efficacy evidence for CHM/medicinal plant-derived ELNVs (Kilasoniya et al., 2023; Orefice et al., 2023; Lin et al., 2024; Liang et al., 2025; Zhang et al., 2025).

### 3.3 Early or indirect indications: IVD, tendon/ligament, and RA-related inflammation

Direct evidence for CHM/medicinal plant-derived ELNVs in intervertebral disc disorders remains limited. Because the IVD is a closed, avascular, and mechanically constrained tissue, future studies should prioritize local retention, tissue penetration, sustained bioactivity, paired ECM readouts, imaging, mechanical assessment, and local safety rather than simply extrapolating from systemic exposure or non-IVD vesicle platforms (Xu et al., 2023; Zeng et al., 2024).

Evidence in tendon or ligament repair is similarly sparse. These tissues are characterized by low perfusion, high mechanical stress, and limited intrinsic healing, meaning that short-term cellular improvements may not predict durable biomechanical recovery. Future studies should focus on retention under mechanical loading, clearance kinetics, collagen I/III balance, scar formation, load-bearing function, and medium-to long-term follow-up (Mao et al., 2024).

RA-related musculoskeletal inflammation provides an immunomodulatory context rather than a structural regeneration model (Fang et al., 2023). Existing evidence mainly supports anti-inflammatory plausibility, including phytomedicine extracts that suppress synovial inflammation and plant-derived EV platforms designed for lesion-selective immune modulation, with PI3K–AKT/NF-κB-related signaling appearing as a recurring mechanistic hub (Chen et al., 2024; Han R. et al., 2025; Yao et al., 2025). However, translation remains limited by rodent model dependence, extract or EV heterogeneity, incomplete PK/BD evidence, and insufficient long-term safety data. These modules should therefore be positioned as hypothesis-generating directions rather than mature orthopaedic indications.

## 4 Mechanistic integration

Across orthopaedic disease modules, CHM/medicinal plant-derived ELNVs are better interpreted as multi-component modulators of coupled pathological networks than as single-target therapeutic agents. The available evidence most consistently converges on three mechanistic domains: bone remodeling and bone–vascular coupling, inflammation–oxidative stress–ECM homeostasis, and cargo- and delivery-dependent biological interpretation. This integrated view is particularly relevant for orthopaedic disorders, in which bone loss, cartilage degeneration, impaired repair, and chronic inflammation often develop through overlapping pathways rather than isolated molecular events (Karabay et al., 2025; Zuo et al., 2025).

### 4.1 Bone remodeling and bone–vascular coupling

In osteoporosis and bone repair-related settings, the most direct mechanistic signal is regulation of the osteogenesis–osteoclastogenesis balance. Reported CHM/medicinal plant-derived ELNVs are commonly associated with enhanced osteogenic differentiation, mineralization, and bone formation markers, including RUNX2 and OCN, together with modulation of osteogenic pathways such as Wnt/β-catenin, MAPK, and PI3K/AKT cascades (Seo et al., 2023; Cao et al., 2024). In parallel, suppression of osteoclastogenic activity is often framed through the RANKL/OPG axis and downstream NF-κB-associated inflammatory signaling, suggesting that these vesicles may rebalance bone remodeling by simultaneously promoting bone formation and restraining bone resorption.

Bone repair and defect-healing contexts further highlight the importance of bone–vascular coupling. In these models, angiogenic cues such as VEGF/VEGFR2 activation and endothelial responses may precede or accompany later osteogenic marker expression, supporting an “angiogenesis first, osteogenesis later” sequence. Therefore, future studies should pair osteogenic readouts, such as RUNX2 and OCN, with vascular indicators, including CD31, endomucin, or H-type vessel-related signals. Such paired endpoints would make mechanistic claims more compatible with the biological sequence of bone regeneration.

### 4.2 Inflammation–oxidative stress–ECM axis

In osteoarthritis, intervertebral disc degeneration, and rheumatoid arthritis-related musculoskeletal inflammation, the most transferable mechanistic axis is the interaction among inflammatory signaling, oxidative stress, and extracellular matrix homeostasis. In cartilage-related models, disease progression is closely associated with increased catabolic mediators such as MMP-13 and ADAMTS-5 and reduced anabolic matrix components such as COL2A1 and Aggrecan. Accordingly, a credible ECM-protective claim should ideally demonstrate both suppression of matrix degradation and preservation or restoration of matrix synthesis, rather than relying on a single marker (You et al., 2023).

Inflammatory and oxidative pathways provide the upstream context for this ECM imbalance. Plant- or CHM-related vesicle systems are frequently linked to inhibition of NF-κB-associated cytokine production, including TNF-α, IL-1β, and IL-6, as well as attenuation of inflammasome-related activation such as NLRP3 (Raimondo et al., 2022; Ou et al., 2023; Ramírez et al., 2024). At the same time, activation of antioxidant defenses, particularly the Nrf2–HO-1 axis and restoration of endogenous antioxidant systems such as SOD, CAT, and GSH, may help reduce ROS-driven tissue damage (Kim et al., 2023; Huang et al., 2025; Karabay et al., 2025). In IVD and tendon/ligament contexts, where direct CHM-ELNV evidence remains sparse, these pathways should be interpreted as mechanistic hypotheses requiring disease-specific validation rather than definitive proof. Similarly, in RA-related musculoskeletal inflammation, PI3K–AKT/NF-κB-related immune modulation may support anti-inflammatory plausibility, but structural protection, joint function, and long-term safety still require independent confirmation.

### 4.3 Cargo causality and delivery-dependent interpretation

The multi-target profile of CHM/medicinal plant-derived ELNVs is likely related to their complex cargo architecture, including small RNAs, proteins, lipids, and phytochemical-associated constituents. Small RNAs or microRNAs may participate in the regulation of differentiation- and inflammation-related genes, whereas lipid and protein components may influence membrane interaction, uptake, endosomal trafficking, mitochondrial stress responses, and immune recognition (Subha et al., 2023). Phytochemical-associated constituents may further contribute to antioxidant and anti-inflammatory effects (Urzì et al., 2022; Chen and Cai, 2023). However, most current evidence remains associative: the presence of a cargo component does not prove that it is necessary or sufficient for the observed orthopaedic phenotype.

Therefore, future mechanistic studies should move from cargo description toward cargo causality. A practical validation strategy would combine multi-omics fingerprinting with perturbation–rescue experiments, including RNase, protease, or lipid-disruption controls; mimic–inhibitor or overexpression–knockdown approaches for candidate RNAs or proteins; and rescue experiments linked to predefined functional endpoints (López de las Hazas et al., 2023; Xu et al., 2023; Kürtösi et al., 2024). For bone-related applications, these endpoints may include RUNX2/OCN expression, mineralization, RANKL/OPG balance, and VEGF-associated angiogenesis. For cartilage or disc-related applications, they should include MMP-13/ADAMTS-5 suppression and COL2A1/Aggrecan preservation. Uptake and intracellular trafficking should also be verified before claiming cross-species or tissue-specific regulatory effects (Garaeva et al., 2021; You et al., 2021).

Finally, mechanistic interpretation must consider the route of administration. Oral delivery is relevant to systemic or gut–bone axis regulation, but requires evidence of gastrointestinal stability, exposure–response relationships, and biodistribution. Local delivery, including intra-articular or defect-site administration, may improve tissue exposure, but requires assessment of retention, clearance, sterility, endotoxin burden, and local inflammatory reactions (Kim et al., 2022; Orefice et al., 2023; Shao et al., 2023). Non-CHM plant vesicles and engineered mammalian EVs can provide useful delivery-design analogies, but they should not be treated as direct efficacy evidence for CHM/medicinal plant-derived ELNVs (Karamanidou and Tsouknidas, 2021; Zhao et al., 2024; Kim et al., 2025). Thus, mechanistic claims should be judged within a combined framework of cargo causality, delivery-dependent exposure, and disease-relevant functional validation.

### 4.4 Distinctive features of CHM/medicinal plant-derived ELNVs

Compared with general edible plant-derived EVs, CHM/medicinal plant-derived ELNVs have several distinctive features that are particularly relevant to orthopaedic translation (Wang et al., 2025). First, their source selection is not based solely on nutritional availability or botanical convenience, but is often supported by traditional pharmacological use and disease-oriented therapeutic rationale (Guo et al., 2023; Liu et al., 2024; Zhang et al., 2024). This provides a useful basis for selecting candidate medicinal plants for bone, cartilage, inflammatory, or regenerative indications. Second, these vesicles may retain a more complex source-related cargo profile, including small RNAs, proteins, lipids, and phytochemical-associated constituents, which may contribute to coordinated regulation of inflammation, oxidative stress, bone remodeling, and extracellular matrix homeostasis (Han X. et al., 2025). Third, their pharmacological complexity may better match the multi-pathway nature of orthopaedic diseases, in which degeneration and repair are rarely controlled by a single molecular target.

However, these advantages should not be interpreted as direct proof of efficacy. In this review, we distinguish direct orthopaedic evidence from CHM/medicinal plant-derived ELNVs, supportive biological plausibility from broader plant-derived EV studies, and transferable delivery or engineering principles from non-CHM or mammalian EV platforms. This distinction helps preserve the thematic focus of the review while avoiding inappropriate extrapolation from general edible plant-derived EVs to CHM/medicinal plant-derived ELNVs.

## 5 Translation and prospects

Although CHM/medicinal plant-derived ELNVs show encouraging preclinical potential, they remain far from being clinically mature orthopaedic therapeutics. The key translational issue is no longer whether these vesicles can produce biological effects in selected models, but whether they can be developed into reproducible, interpretable, and safety-supported products (Cong et al., 2022). Recent reviews suggest that the field is still moving from exploratory proof-of-concept studies toward standardized translational development (Urzì et al., 2021; Wang et al., 2022; Yingqi et al., 2025). Three barriers are particularly important: CMC and batch consistency, potency and cargo causality, and PK/BD-supported long-term safety. It should also be noted that the current literature remains largely oriented toward positive outcomes, whereas negative or neutral findings, failed delivery designs, dose-limiting toxicity, and irreproducible results are rarely reported. This imbalance may contribute to publication bias and overestimation of translational feasibility.

### 5.1 CMC and batch consistency

The first barrier is chemistry, manufacturing, and controls (CMC)-oriented quality consistency. CHM/medicinal plant-derived ELNVs are influenced by plant species, medicinal part, cultivation environment, harvest season, storage, processing, and extraction workflow. These variables may change particle yield, purity, cargo composition, and biological activity. Therefore, future translational studies should move from descriptive preparation toward source–process–product control. Source authentication, raw-material quality, isolation parameters, storage conditions, and release criteria should be predefined whenever possible. For CHM/medicinal plant-derived ELNVs, risk profiling should also include microbial contamination, endotoxin burden, pesticide residues, heavy metals, and other route-dependent safety concerns (Kameli et al., 2021; Woith et al., 2021; Zhao et al., 2024). Without such CMC-oriented documentation, positive preclinical findings will remain difficult to compare, reproduce, or translate.

### 5.2 Potency and cargo causality

The second barrier is the lack of stable potency assays and cargo-causality validation. Many studies report improvements in osteogenic differentiation, inflammatory cytokines, oxidative stress, or ECM markers, but these outcomes are often descriptive and not always linked to defined product attributes. A clinically meaningful potency assay should be mechanism-linked, disease-relevant, and scalable. For bone-related indications, candidate potency readouts may include RUNX2/OCN expression, mineralization, RANKL/OPG balance, or angiogenesis–osteogenesis coupling. For cartilage or disc-related applications, paired readouts such as MMP-13/ADAMTS-5 suppression and COL2A1/Aggrecan preservation are more informative than isolated markers.

Cargo causality remains another unresolved issue. CHM/medicinal plant-derived ELNVs contain small RNAs, proteins, lipids, and phytochemical-associated constituents, but the contribution of each component is rarely proven. Future studies should combine multi-omics fingerprinting with perturbation–rescue experiments, such as RNase, protease, or lipid-disruption controls; mimic–inhibitor or knockdown–rescue designs; and functional validation linked to predefined orthopaedic endpoints (Cao et al., 2023; Hao et al., 2024; Feng et al., 2025). Such designs would help determine whether a candidate cargo is necessary, sufficient, or only correlative, and would also help distinguish true vesicle-mediated effects from effects driven by co-isolated phytochemicals or nonspecific nanoparticle properties.

### 5.3 PK/BD and long-term safety

The third barrier is insufficient pharmacokinetic, biodistribution, and long-term safety evidence. Oral delivery may be attractive for osteoporosis or gut–bone axis regulation, but gastrointestinal degradation, variable intestinal uptake, first-pass metabolism, and reticuloendothelial clearance can strongly influence exposure. Local delivery, including intra-articular or defect-site administration, may increase tissue exposure but introduces different concerns, including retention time, clearance, local immune reaction, sterility, and endotoxin burden. Therefore, PK/BD evaluation should be incorporated early rather than treated as a late-stage regulatory requirement (Fang and Liu, 2022; Wang et al., 2024). Route-specific PK/BD evidence is also necessary for comparing oral, local, and biomaterial-assisted strategies under clinically relevant dosing conditions (Shao et al., 2023; Li et al., 2024).

Long-term safety is especially important for chronic orthopaedic disorders, where repeated administration may be required. Current models often capture short-term improvements but do not adequately reflect aging, mechanical loading, chronic inflammation, multimorbidity, or cumulative toxicity. In the near term, CHM/medicinal plant-derived ELNVs may be more realistic as adjuvant-style, local, topical, functional-food-related, or material-assisted platforms than as stand-alone disease-modifying drugs (Zheng et al., 2025). Progress toward clinical investigation will depend on whether future studies can report not only efficacy, but also reproducibility, dose–response relationships, failure modes, safety margins, and clinically interpretable release criteria (Lian et al., 2022; Nemati et al., 2022).

## 6 Conclusion

CHM/medicinal plant-derived ELNVs represent a promising but still early-stage nanoplatform for orthopaedic diseases. Their main appeal lies in their multi-component cargo architecture and potential ability to coordinate inflammation, oxidative stress, ECM remodeling, bone homeostasis, and bone–vascular coupling. Among current indications, osteoporosis has the strongest preclinical support, whereas osteoarthritis and bone repair remain emerging local-delivery fields, and intervertebral disc disorders, tendon/ligament injury, and RA-related musculoskeletal inflammation are still largely hypothesis-generating.

Future progress should shift from simply reporting positive biological effects to building reproducible and clinically interpretable evidence chains. Priority should be given to source authentication, standardized isolation and characterization, disease-relevant potency assays, cargo-causality validation, route-specific PK/BD assessment, and long-term safety evaluation. Only through such integrated methodological and translational frameworks can CHM/medicinal plant-derived ELNVs move from encouraging preclinical observations toward credible orthopaedic applications.
