# Supplementary material for: Chinese herbal medicine-derived exosome-like nanovesicles for orthopaedic diseases: evidence, methods, and translation
Source: Front Cell Dev Biol. 2026 Jul 10;14:1853729. doi: 10.3389/fcell.2026.1853729 (PMC13395924; doi:10.3389/fcell.2026.1853729)
Supplement: Supplementary file 1 [file Supplementaryfile1.pdf]

Supplementary Material

1.1 Supplementary Figures

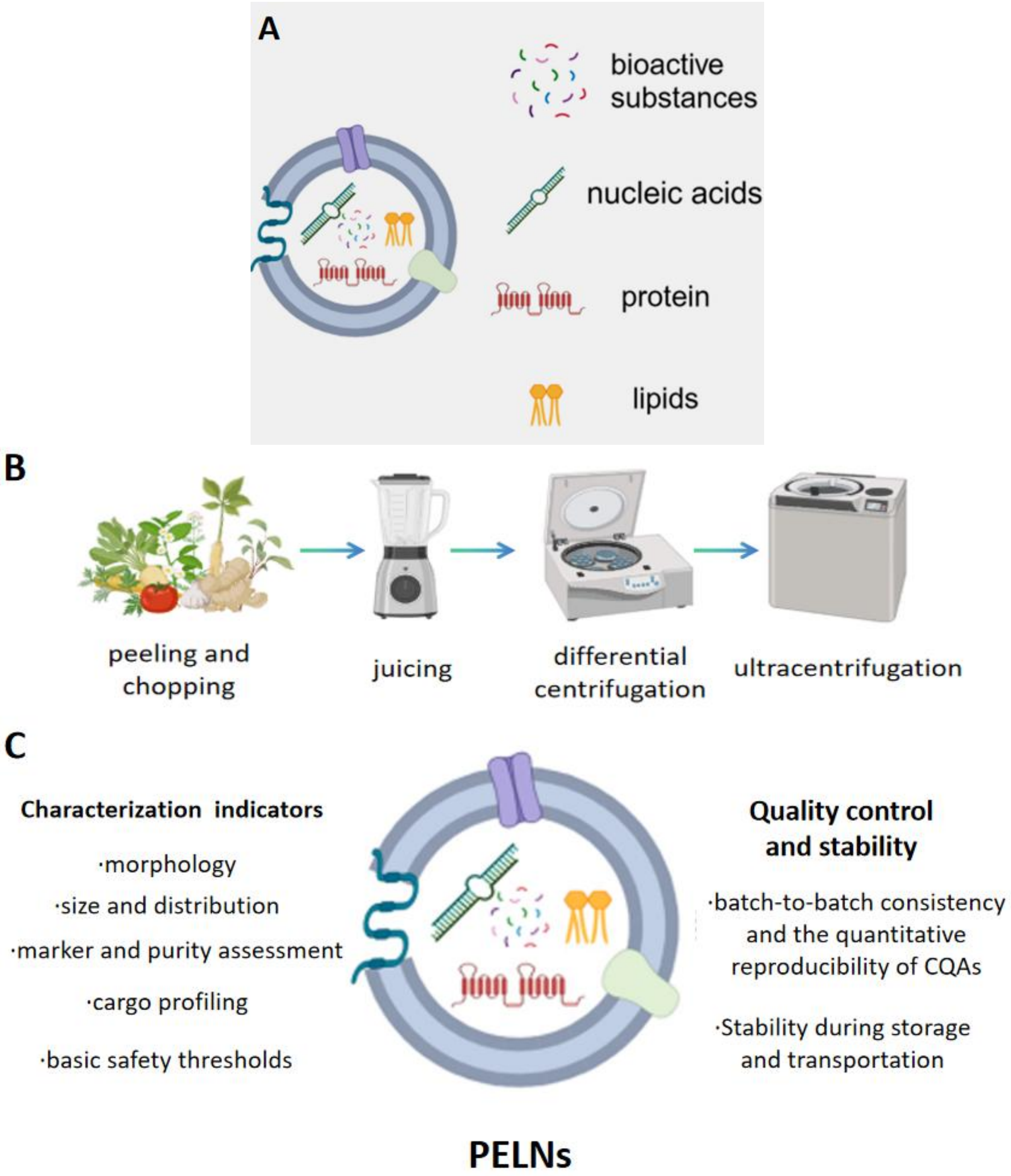
A
bioactive substances
nucleic acids
protein
lipids
B
peeling and chopping
juicing
differential centrifugation
ultracentrifugation
C
Characterization indicators
·morphology
·size and distribution
·marker and purity assessment
·cargo profiling
·basic safety thresholds
Quality control and stability
·batch-to-batch consistency and the quantitative reproducibility of CQAs
·Stability during storage and transportation
PELNs

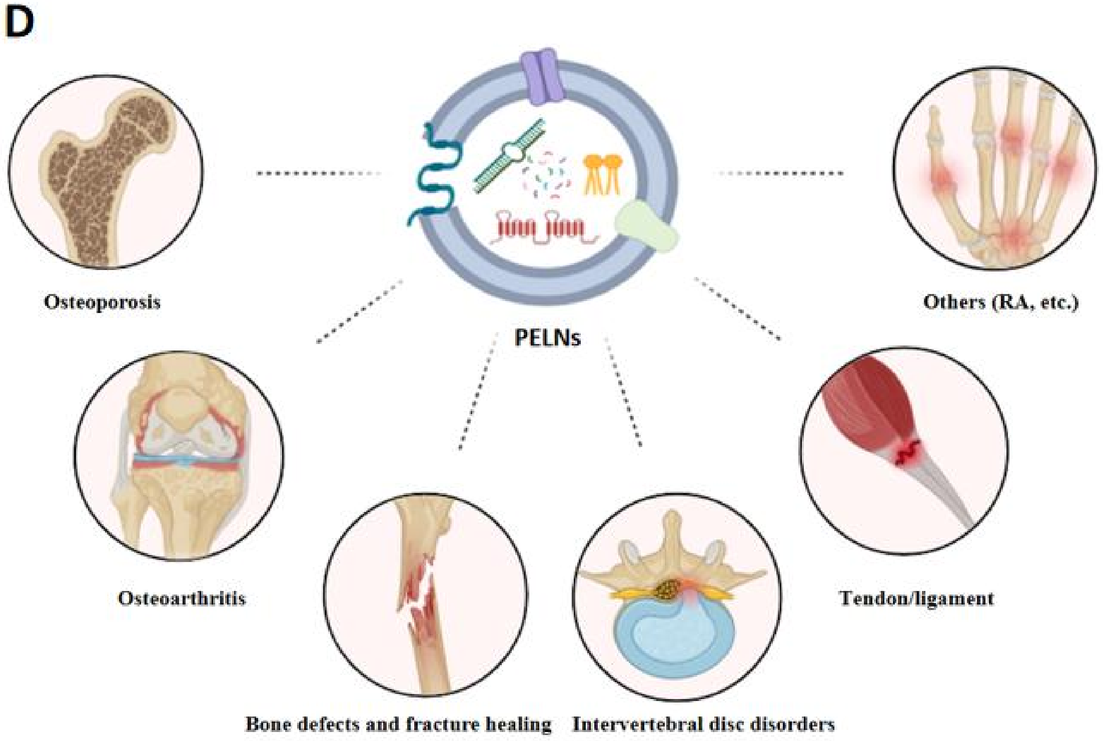

Figure 1. Overview of plant-derived exosome-like nanovesicles and their potential applications in orthopaedic diseases.

(A) Main components of PELNs, including nucleic acids, proteins, lipids, and small bioactive substances, which together contribute to their biological functions and therapeutic potential.(B) General workflow for the isolation of PELNs.(C) Characterization and quality-control considerations for PELNs.(D) Overview of the potential applications of PELNs in orthopaedic diseases, including osteoporosis, osteoarthritis, bone defects and fracture healing, intervertebral disc degeneration, tendon injury, and rheumatoid arthritis.

1.2 Supplementary tables

Table 1. Comparative appraisal of evidence maturity and translational readiness of CHM/medicinal plant-derived ELNVs across orthopaedic disease modules

Disease module	Evidence maturity	Key readouts	Main gaps	Translational priority
**Osteoporosis**	Relatively highest	Osteogenesis–osteoclastogenesis balance; RUNX2/OCN; mineralization; RANKL/OPG; gut-bone or immune-metabolic signals.	Variable characterization; incomplete cargo causality, PK/BD, dosing, and endpoint standardization.	Priority module for standardized preclinical validation; strongest current orthopaedic evidence base.
**Osteoarthritis**	Early to moderate	ECM synthesis-degradation balance; MMP-13/ADAMTS-5; COL2A1/Aggrecan; inflammatory cytokines; structural or pain endpoints.	Limited direct CHM/medicinal plant-derived ELNV evidence; insufficient intra-articular retention, local safety, PK/BD, and cargo causality.	Promising local-delivery field, but disease-modifying claims should remain cautious.

Disease module	Evidence maturity	Key readouts	Main gaps	Translational priority
**Bone defect/fracture healing**	Moderate and emerging	Angiogenesis-osteogenesis coupling; RUNX2/OCN; CD31/endomucin or H-type vessel signals; mineralized tissue formation.	Incomplete long-term remodeling data, scaffold/product characterization, full PK/BD, and cargo-function validation.	Strong rationale for biomaterial-assisted local regeneration studies.
**Intervertebral disc disorders**	Low	ECM homeostasis; MMP-13/ADAMTS-5; COL2A1/Aggrecan; oxidative or mitochondrial stress; imaging and mechanical assessment.	Direct evidence is scarce; intradiscal retention, penetration, local safety, PK/BD, and cargo causality are not established.	Exploratory, hypothesis-generating direction.
**Tendon/ligament injury**	Low	Collagen I/III balance; scar formation; inflammatory control; load-bearing and biomechanical recovery.	Direct CHM/medicinal plant-derived ELNV evidence is lacking; mechanical-loading validation, retention kinetics, and long-term functional endpoints are insufficient.	Future exploratory module requiring mechanically relevant models.
**RA-related musculoskeletal inflammation**	Early to moderate	Synovial inflammation; macrophage M1/M2 polarization; TNF-α, IL-1β, IL-6; joint swelling or arthritis scores.	More immunomodulatory than structural-regenerative; limited long-term safety, PK/BD, and cargo-specific mechanisms.	Relevant inflammatory musculoskeletal module; translational claims should be conservative.

Note: Evidence maturity refers to the relative strength of the preclinical evidence package rather than clinical readiness. It was qualitatively judged by considering direct CHM/medicinal plant-derived ELNV evidence, in vivo/model support, methodological characterization, PK/BD availability, cargo-causality validation, and translational depth. Direct CHM/medicinal plant-derived ELNV evidence should be distinguished from broader plant-derived vesicle evidence and engineered mammalian EV analogies.

Abbreviations: BD, biodistribution; CHM, Chinese herbal medicine; ECM, extracellular matrix; ELNVs, exosome-like nanovesicles; EVs, extracellular vesicles; OCN, osteocalcin; PK, pharmacokinetics; RA, rheumatoid arthritis.
